# A Minimally Invasive Method for Observing Wind-Up of Flexion Reflex in Humans: Comparison of Electrical and Magnetic Stimulation

**DOI:** 10.3389/fnins.2022.837340

**Published:** 2022-02-23

**Authors:** Tomoya Taniguchi, Tomoaki Alex Kinukawa, Nobuyuki Takeuchi, Shunsuke Sugiyama, Makoto Nishihara, Kimitoshi Nishiwaki, Koji Inui

**Affiliations:** ^1^Department of Anesthesiology, Nagoya University Graduate School of Medicine, Nagoya, Japan; ^2^Neuropsychiatric Department, Aichi Medical University, Nagakute, Japan; ^3^Department of Psychiatry and Psychotherapy, Gifu University, Gifu, Japan; ^4^Multidisciplinary Pain Center, Aichi Medical University, Nagakute, Japan; ^5^Department of Functioning and Disability, Institute for Developmental Research, Aichi Developmental Disability Center, Kasugai, Japan; ^6^Department of Integrative Physiology, National Institute for Physiological Sciences, Okazaki, Japan

**Keywords:** central sensitization, flexion reflex, magnetic stimulation, *N*-methyl-D-aspartate receptor, short-term plasticity, temporal summation, wind-up, withdrawal reflex

## Abstract

Wind-up like pain or temporal summation of pain is a phenomenon in which pain sensation is increased in a frequency-dependent manner by applying repeated noxious stimuli of uniform intensity. Temporal summation in humans has been studied by observing the increase in pain or flexion reflex by repetitive electrical or thermal stimulations. Nonetheless, because the measurement is accompanied by severe pain, a minimally invasive method is desirable. Gradual augmentation of flexion reflex and pain induced by repetitive stimulation of the sural nerve was observed using three stimulation methods—namely, bipolar electrical, magnetic, and monopolar electrical stimulation, with 11 healthy male subjects in each group. The effects of frequency, intensity, and number of repetitive stimuli on the increase in the magnitude of flexion reflex and pain rating were compared among the three methods. The reflex was measured using electromyography (EMG) from the short head of the biceps femoris. All three methods produced a frequency- and intensity-dependent progressive increase in reflex and pain; pain scores were significantly lower for magnetic and monopolar stimulations than for bipolar stimulation (*P* < 0.05). The slope of increase in the reflex was steep during the first 4–6 stimuli but became gentler thereafter. In the initial phase, an increase in the reflex during the time before signals of C-fibers arrived at the spinal cord was observed in experiments using high-frequency stimulation, suggesting that wind-up was caused by inputs of A-fibers without the involvement of C-fibers. Magnetic and monopolar stimulations are minimally invasive and useful methods for observing the wind-up of the flexion reflex in humans. Monopolar stimulation is convenient because it does not require special equipment. There is at least a partial mechanism underlying the wind-up of the flexion reflex that does not require C-fibers.

## Introduction

Wind-up or temporal summation of pain is a phenomenon in which pain or firing of spinal dorsal horn neurons is increased in a frequency-dependent manner by applying repetitive noxious stimuli (Mendell and Wall, 1965; Herrero et al., 2000). Some of the mechanisms of wind-up share those of central sensitization, and are thought to be involved in hyperalgesia or chronic pain (Li et al., 1999). Therefore, investigation of the wind-up phenomenon would be useful to investigate certain aspects of pathological pain conditions.

In animal studies, wind-up has been observed as facilitated firing of spinal dorsal horn neurons by repetitive electrical stimulation of peripheral C-fibers (Mendell, 1966) or an increase in the electromyogram of the flexion or withdrawal reflex by repetitive electrical stimulation (Price, 1972). In humans, wind-up has been indirectly studied by observing the increase in pain sensation or magnitudes of the RIII component of the flexion reflex by repetitive electrical or heat stimulation (Arendt-Nielsen et al., 1994; Vierck et al., 1997; Guirimand et al., 2000; Terry et al., 2011). The flexion reflex is stably recorded and reproducible (Willer, 1985). Because the short-term plasticity of pain can be easily and clearly observed in humans, wind-up of the flexion reflex is considered a useful method for understanding the pathophysiology of pain. However, it is not widely used because the measurement is accompanied by severe pain, and only a few studies have been conducted using the method. Therefore, less invasive methods are desired.

In this study, we aimed to establish a minimally invasive method for observing wind-up of the flexion reflex in humans and to make it more commonly available. For this purpose, the effects of repetitive stimulation on flexion reflex and pain were compared among three stimulation methods—namely, magnetic stimulation, bipolar electrical stimulation, and monopolar electrical stimulation. Generally, temporal summation of pain in humans is not considered identical to the wind-up of dorsal horn neurons or flexion reflexes in animal experiments. However, to simplify the expression, we used the term “wind-up” in this study to denote progressive increases in the reflex magnitude and pain.

## Materials and Methods

The study was conducted on 33 healthy male volunteers (age, 21–57 years; mean, 33.1 years). None of the participants had a history of neurological or pain disorders or substance abuse in the last 2 years. They were free of medication at testing. This study was conducted in accordance with the principles embodied in the Declaration of Helsinki and was approved by the Ethics Committee of the National Institute for Physiological Sciences, Okazaki, Japan (approval number: 21A001). Written informed consent forms were obtained from all participants.

### Recordings

**FIGURE 1:**
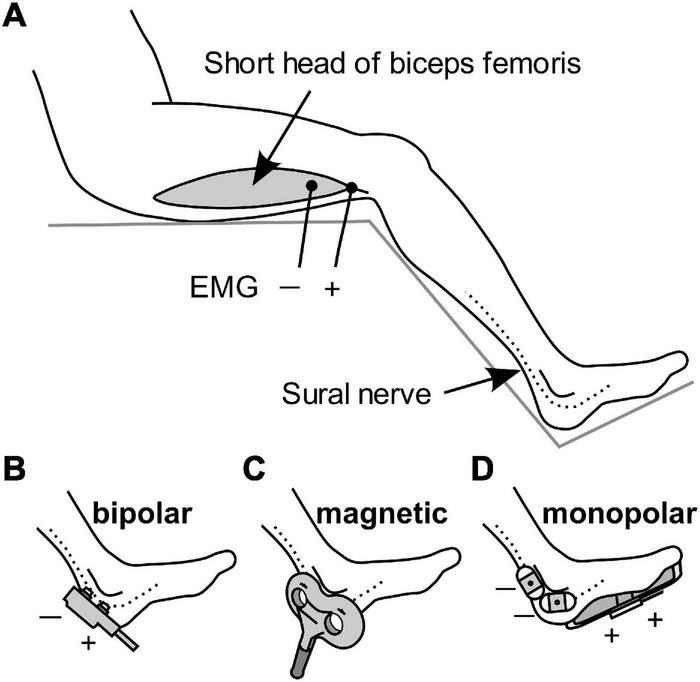
Schematic diagram of the stimulation methods and EMG recordings. **(A)** Electromyography (EMG) was used for recording flexion reflexes from the biceps femoris. **(B–D)** Stimulation of the sural nerve by bipolar electrical stimulation, transcutaneous magnetic stimulation, and monopolar electrical stimulation.

The participants were randomly assigned to three different groups receiving stimulation with different methods, with 11 participants in each group. All stimuli were applied to the sural nerve at the level of the lateral malleolus, and flexion reflexes were recorded from the short head of the biceps femoris muscle, ipsilateral to the stimulation, using surface electromyography (EMG). Ag/AgCl disk recording electrodes were attached to the muscle belly as the cathode and to the tendon as the anode with a 5-cm separation (Figure 1A). A band ground electrode was placed midway between the stimulation and the recording electrodes. EMG signals were amplified, filtered (10–2 kHz), and stored (−100 to 400 ms) at a sampling rate of 10 kHz using an EMG/evoked potential measuring system (MEB-2300, Nihon Kohden, Tokyo, Japan). The obtained EMG waveforms were full-wave rectified and the average value in the interval from -100 to 0 ms was subtracted as a direct-current offset. The area under the curve (AUC) in the interval of 70–200 ms after the onset of stimulation was calculated as the magnitude of the RIII component of the flexion reflex. Although this procedure excluded the RII component appearing at 40–60 ms, its occurrence was very rare in this study.

### Stimulation Methods

The first group received **bipolar electrical stimulation** with a bipolar electrode (NM-420S, Nihon Kohden) with two felt tips, each of 8 mm diameter, separated by 23 mm. The electrode was placed on the skin and sural nerve, with the anode in the distal position (Figure 1B). The stimulus was a rectangular 1-ms single pulse.

Stimulation in the second group was *via* transcutaneous **magnetic stimulation** (Figure 1C), which was delivered by Magstim Super Rapid device (The Magstim Company, Whitland, United Kingdom). TMS is generally non-invasive. The stimulation output site of the 8-shaped probe was placed on the sural nerve. The duration of the biphasic waveform was less than 1 ms.

The third group was subjected to **monopolar electrical stimulation** (Figure 1D). Two Ag/AgCl electrodes with adhesive conductive gel (Vitrode F 25 mm × 45 mm; Nihon Kohden) were attached to the skin over the sural nerve as the cathode. The anode was a counter electrode with a conductive adhesive gel (180 mm × 115 mm, Valleylab Polyhesive Patient Return Electrode E7507; Covidien, Mansfield, MA, United States) attached to the sole of the foot. The stimulus was a rectangular 1-ms single pulse.

### Procedures

In each stimulation group, three experiments were performed with the following procedures: First, the experimental procedures were explained briefly, emphasizing that participants could terminate the experiment at any time if the pain was intolerable. Thereafter, the participants sat with their hip and knee angles at approximately 90° and 130°, respectively.

The pain and reflex thresholds were determined at the beginning. The stimulus was delivered at 0.3 Hz and the intensity was gradually increased. Using an up-and-down procedure, the reflex threshold was determined as the intensity at which the flexion reflex was elicited by 50% of the stimulations. The pain threshold was defined as the intensity at which the tactile sensation turned to pain. Thereafter, three experiments were conducted in random order. There was a 1-minute interval between measurements.

#### Effects of Stimulus Number (Experiment 1)

The intensity was fixed at the reflex threshold, and 20 consecutive stimuli were applied at 2 Hz. Measurements were taken five times and the average value was calculated. The magnitude of the AUC was plotted against the stimulus number. A segmented linear regression analysis was performed using the segmented package for R (version 4.0.5) (Muggeo, 2017) and the point of the slope change (break point) was determined.

#### Effects of Stimulation Frequency (Experiment 2)

The stimulation frequency was set at 0.5, 1, 2, 3, 4, or 5 Hz. At each frequency, 10 consecutive reflex threshold stimuli were delivered. The measurement was performed twice with a different order and the mean values were compared.

#### Effects of Intensity (Experiment 3)

The stimulation intensity was set at 0.5, 0.6, 0.7, 0.8, 0.9, or 1.0 times the reflex threshold. For each stimulation intensity, 10 consecutive stimuli were delivered at 2 Hz. The AUC was compared among the six intensities.

For all measurements, pain scores for the first and last stimuli in a series of consecutive stimuli were recorded using a Numerical Rating Scale. The pain score was defined as follows: 0, no pain at all; and 100, maximum possible pain.

### Statistical Analysis

The sample size was calculated to expect the detection of an interaction between stimulation methods and the wind-up effect in pain scores, using G power (version 3.1), with 33 participants (effect size *f* = 0.25, α = 0.05, power = 0.8, correlation among repeated measures = 0.65). For each experiment, the AUC values and pain scores were compared among three methods (stimulation), conditions (intensity or frequency), or stimulus number (wind-up) using two-way or three-way repeated measures analysis of variance (ANOVA) using SPSS 27.0 (IBM Corp, Armonk, NY, United States). For *post-hoc* paired comparisons, a Bonferroni correction was applied. Statistical significance was set at *P* < 0.05.

## Results

### Flexion Reflex

**FIGURE 2:**
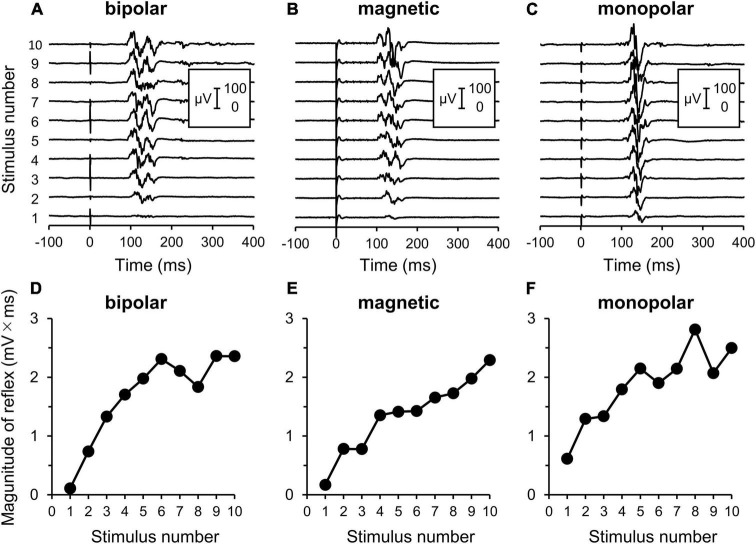
Electromyography (EMG) recordings of flexion reflex and procedures of analyses. **(A–C)** Original waveforms of a representative subject elicited by a series of 10 consecutive stimuli at the reflex threshold at 2 Hz by bipolar, magnetic, and monopolar stimulations in Experiment 3. **(D–F)** Relationship between the reflex magnitude [area under the curve (AUC)] and stimulus number. Note that the reflex is clearly enhanced with repeated stimulations.

Some measurements could not be completed because while one participant in the bipolar group experienced intolerable pain, reflex could not be elicited even at the maximal stimulation intensity in two participants in the magnetic group. Therefore, three additional subjects were enrolled so that each stimulation group had 11 subjects. An example of the measurement is shown in Figure 2.

#### Experiment 1. Effects of Stimulus Number

**FIGURE 3:**
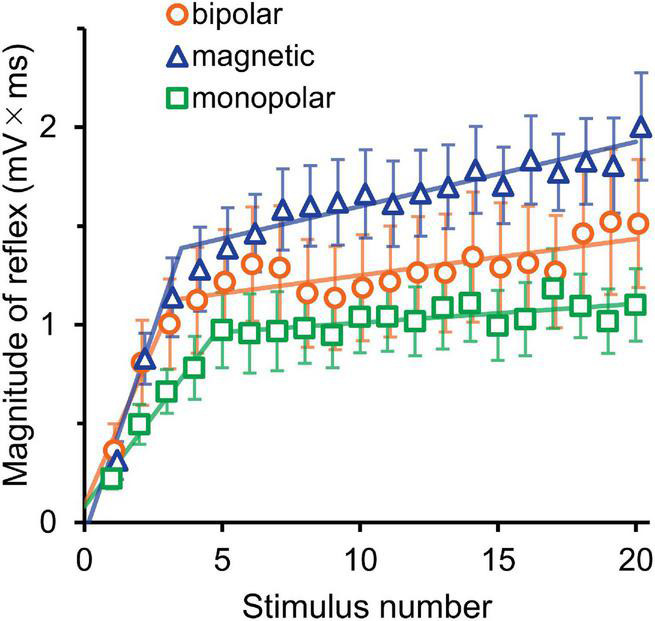
Experiment 1. The mean magnitude ± SE of the reflex for 20 consecutive stimulations is plotted against the stimulus number. The lines show the results of segmented linear regression analysis.

The averaged waveforms are shown in Supplementary Figure 1. Figure 3 presents the mean magnitudes of reflexes and the results of their analysis by segmented linear regression. The results of two-way ANOVA (stimulation × wind-up) indicated that the stimulation effect was not significant (*F*_2,30_ = 1.8, *P* = 0.190). The wind-up effect of the consecutive stimuli was evident (*F*_19,570_ = 37.4, *P* = 2.8 × 10^–87^, partial η^2^ = 0.56). In the segmented regression model, the median (interquartile range) of the breakpoint was 3.3 (2.4–3.9), 6.0 (3.4–7.4), and 5.0 (3.5–8.0) for bipolar, magnetic, and monopolar stimulations, respectively.

#### Experiment 2. Effects of Stimulation Frequency

**FIGURE 4:**
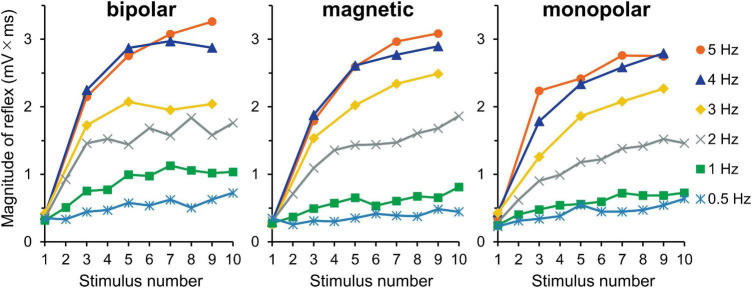
Experiment 2. Mean reflex magnitude for 10 consecutive stimulations at six different stimulation frequencies. Although the wind-up effect is present for all frequency conditions, it is more obvious at higher frequencies.

The averaged waveforms are shown in Supplementary Figure 2. Figure 4 presents the mean magnitudes of reflexes. Owing to the very short stimulation intervals at 3, 4, and 5 Hz, the recording equipment was unable to pick up the triggers and record the EMG of the even-numbered stimuli; nevertheless, the 10 consecutive stimuli themselves were produced without any problem. Thus, the data with odd stimulus number were used for the analysis. The results of three-way ANOVA (stimulation × frequency × wind-up) showed that the effect of stimulation was not significant (*P* = 0.717), and no interaction was detected. However, wind-up (*F*_4,116_ = 120.5, *P* = 2.3 × 10^–40^, partial η^2^ = 0.81) and frequency (*F*_5,145_ = 107.6, *P* = 5.4 × 10^–47^, partial η^2^ = 0.79) were significant. The comparisons between frequencies showed significant difference for all pairs, except for the 4–5 Hz pair. The frequency × wind-up interaction was significant (*F*_20,580_ = 35.0, *P* = 4.2 × 10^–86^, partial η^2^ = 0.55). When wind-up was tested for each stimulation frequency, all frequencies significantly affected the reflex magnitude.

#### Experiment 3. Effects of Intensity

**FIGURE 5:**
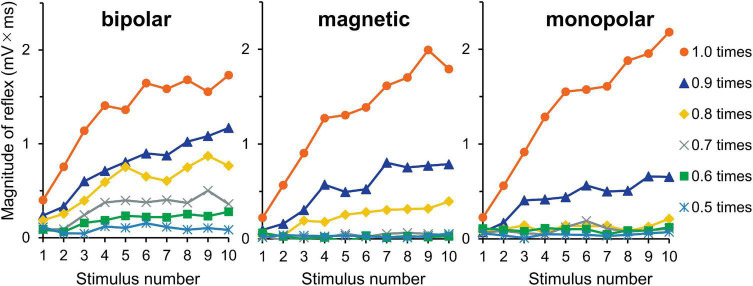
Experiment 3. The reflex magnitude for 10 consecutive stimulations at six stimulation intensities.

The averaged waveforms are shown in Supplementary Figure 3. Figure 5 shows the mean magnitudes. The results of three-way ANOVA (stimulation × intensity × wind-up) showed that intensity (*F*_5,150_ = 67.3, *P* = 1.3 × 10^–36^, partial η^2^ = 0.69) and wind-up (*F*_9,270_ = 42.6, *P* = 6.0 × 10^–47^, partial η^2^ = 0.59) were significant factors, but stimulation was not (*P* = 0.146). The intensity × wind-up interaction was significant (*F*_45,1350_ = 21.2, *P* = 8.4 × 10^–125^, partial η^2^ = 0.41). *Post-hoc* paired comparisons showed that the magnitude was significantly different among the 10 stimuli only for 0.9 (*P* = 6.8 × 10^–4^) and 1.0 (*P* = 2.9 × 10^–6^) times the reflex threshold. When the increment of the reflex magnitude between the first and tenth stimuli was compared between the 0.9- and 1.0-times conditions (stimulus × AUC increment), the wind-up effect was significantly greater for the 1.0-times condition (*P* = 1.6 × 10^–5^). The results for the increments between the first and fifth stimuli were similar (*P* = 1.2 × 10^–6^).

### Pain Rating

**FIGURE 6:**
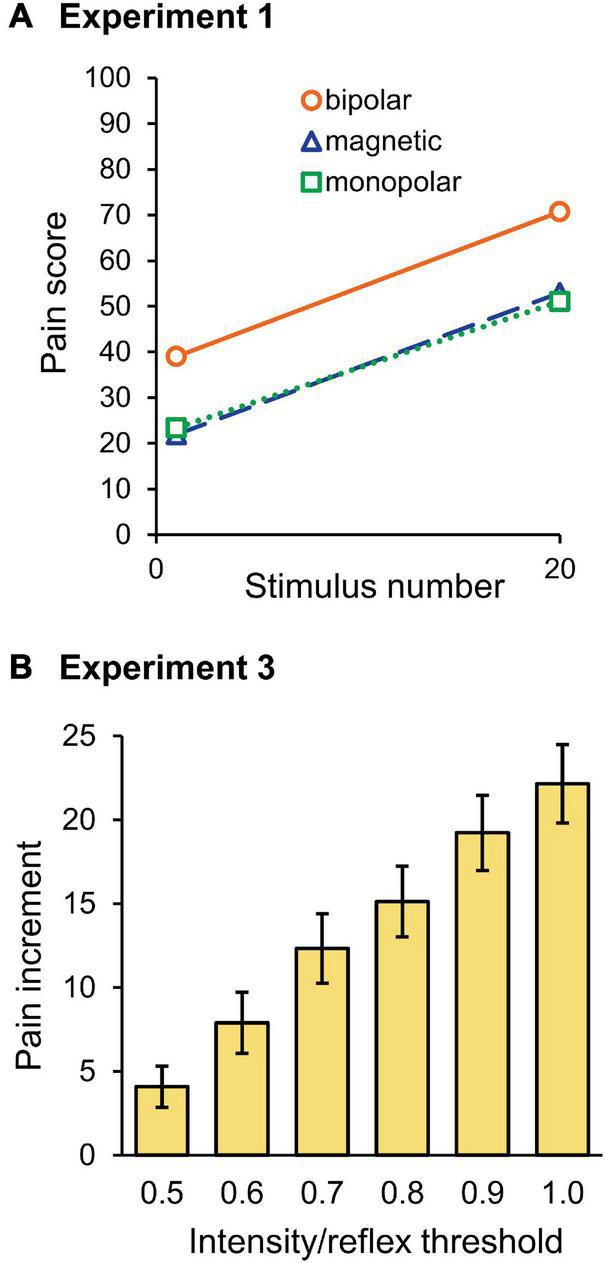
Pain ratings. **(A)** Mean score of the pain rating scale for the first and last stimuli in Experiment 1. Note that the pain score for bipolar stimulation was significantly higher than for magnetic or monopolar stimulation, however, the wind-up effect was not significantly different among the three groups. **(B)** Mean pain score increment between the first and last stimuli in Experiment 3. With stronger stimulation intensities, the pain score increase was greater.

The mean pain score in Experiment 1 is shown in Figure 6A. The score for the last stimulus was significantly greater than that for the first stimulus (*F*_1,30_ = 83.0, *P* = 3.8 × 10^–10^, partial η^2^ = 0.74). Although stimulation was a significant factor in determining the pain score (*F*_2,30_ = 5.34, *P* = 0.010, partial η^2^ = 0.26), the stimulation × wind-up interaction was not (*P* = 0.875), that is, the pain score for bipolar stimulation was significantly greater than that for magnetic (*P* = 0.026) or monopolar (*P* = 0.024) stimulation, but their wind-up effects were not different. There was no significant difference in the pain score between the magnetic and monopolar groups (*P* > 0.999). In Experiment 2, frequency (*F*_5,145_ = 96.9, *P* = 1.9 × 10^–44^, partial η^2^ = 0.77), wind-up (*F*_1,29_ = 145.7, *P* = 7.8 × 10^–13^, partial η^2^ = 0.83), and stimulation (*F*_2,29_ = 10.5, *P* = 3.7 × 10^–4^, partial η^2^ = 0.42) significantly affected the pain score. The overall pain score for bipolar stimulation was also greater than that of magnetic (*P* = 5.0 × 10^–4^) and monopolar (*P* = 0.004) ones. *Post-hoc* paired *t*-tests showed that wind-up effects were significant at all stimulation frequencies (*P* = 2.2 × 10^–14^–8.6 × 10^–7^). In Experiment 3, all three factors significantly affected the pain score: intensity (*F*_5,145_ = 135.8, *P* = 7.0 × 10^–53^, partial η^2^ = 0.82), wind-up (*F*_1,29_ = 67.2, *P* = 4.9 × 10^–9^, partial η^2^ = 0.70), and stimulation (*F*_2,29_ = 22.0, *P* = 1.5 × 10^–6^, partial η^2^ = 0.60). The intensity × wind-up interaction was significant (*F*_5,145_ = 29.6, *P* = 1.19 × 10^–20^), and *post-hoc* tests revealed that the pain score was significantly greater for the last stimulus at all intensities from 0.5 to 1.0 times the reflex threshold (*P* < 0.003). Similar to other experiments, the overall pain score for bipolar stimulation was greater than that for magnetic (*P* = 1.0 × 10^–5^) and monopolar (*P* = 1.0 × 10^–5^) stimulations.

Because the stimulation intensity would have affected the first stimulus pain in Experiment 3, the increment in the pain score between the first and tenth stimuli was compared among the six intensities. As shown in Figure 6B, the pain increment gradually increased as the stimulation intensity increased. The results of two-way ANOVA (stimulation × intensity) showed that intensity (*F*_5,145_ = 29.6, *P* = 1.2 × 10^–20^, partial η^2^ = 0.51) significantly affected the pain increment. *Post-hoc* tests showed that the difference was significant for all pairs, except for the pairs with 0.6–0.7, 0.7–0.8, and 0.9–1.0 times the threshold. Therefore, not only the pain score itself but also the degree of pain increment increased with the increase in stimulation intensity.

## Discussion

We compared bipolar, magnetic, and monopolar stimulations to establish an appropriate method to observe wind-up. The results indicated that each method exerted a frequency- and intensity-dependent progressive increase in reflex and pain. Pain sensation was significantly weaker for magnetic and monopolar stimulations than for bipolar stimulation.

### Nature of the Response Augmentation

Optimal stimulation frequencies to evoke wind-up phenomenon are approximately 0.5–3 Hz, as was shown for the reflex in humans (Arendt-Nielsen et al., 1994; Terry et al., 2011) and animals (Schouenborg and Sjolund, 1983) and for spinal neurons in animals (Mendell, 1966). Here, wind-up effects were observed for all conditions from 0.5 to 5 Hz. For stimulation intensity, reflex wind-up was observed at 0.9 and 1.0 times the reflex threshold, with greater effect in the 1.0 times the reflex threshold (Figure 5) as with a previous study, showing that wind-up is dependent upon stimulation intensity (Arendt-Nielsen et al., 2000). This study’s facilitation matches the wind-up concept originally used for spinal nociceptive neurons (Mendell and Wall, 1965).

As shown in Figure 3, reflex magnitude increased steeply after repetitive stimulation approximately up to the fifth stimulus, followed by a more gradual increase. This may suggest that the wind-up is mediated by two different mechanisms. As for the initial increase, the wind-up effect was only mediated by A-fiber input, because the conduction velocity of C-fibers was so slow that signals evoked by the first stimulus did not reach the spinal cord, even when the third reflex occurred by 5 Hz stimulation (Experiment 2). Mechanisms underlying the later phase were uncertain but may include the influence of a ceiling effect, descending inhibitory controls, facilitation by supraspinal mechanisms, C-fiber inputs, and muscle tension. In studies using microneurography, wind-up caused by C-fibers usually shows steep enhancement during initial stimuli, after which the response gradually declines due to a progressive delay in the conduction velocity of C-fibers (Schmelz et al., 1995). Therefore, it is possible that the later phase was due to the gradual decline of the C-fiber activities. However, the present study does not provide direct evidence that C-fiber signals contributed to the wind-up of the reflex.

Overall, pain rating results were comparable to reflex results, consistent with previous studies on temporal pain summation (Vierck et al., 1997; Nielsen and Arendt-Nielsen, 1998; Arendt-Nielsen et al., 2000; Farrell and Gibson, 2007; Terry et al., 2011). Experiment 3 showed that wind-up effect significantly affected pain in all conditions from 0.5 to 1.0 times the reflex threshold, while the reflex wind-up was significant only for 0.9 and 1.0 times. This is because temporal pain summation is sufficiently induced by pain threshold intensity, which was lower than the reflex threshold. In previous studies using radiant or contact heat stimulation, temporal pain summation was observed for both the first and second pain, with clear dominance for the second pain (Vierck et al., 1997; Nielsen and Arendt-Nielsen, 1998). However, in this study, the pain sensation was not temporally separable in all subjects, suggesting a modest contribution of C-fibers, if any. Therefore, Aδ-fibers were probably responsible for pain wind-up.

### Methodological Considerations

The flexion reflex in humans has been studied by applying a train of electrical pulses to the sural nerve (Kugelberg et al., 1960; Shahani and Young, 1971; De Willer, 1977; Sandrini et al., 1993, 2005). To the best of our knowledge, no previous study has used other methods to evoke wind-up following sural nerve stimulation. Due to the possibility of a wind-up effect within the train stimulus, a 1-ms single pulse was used in this study. The RIII reflex is known to be suppressed by the occurrence of RII (Hugon, 1973; De Willer, 1977); however, unlike a train, single pulses rarely evoke the RII reflex and make the RIII reflex clearer and facilitate the calculation of the response.

Magnetic stimulation directly activates the nerves with a minimal effect of the skin, thus the stimulation is non-invasive and almost painless (Panizza and Nilsson, 2005; Kizilay et al., 2011). However, in this study, the RIII reflex with obvious pain sensations was caused by magnetic stimulation. Because afferents responsible for the RIII reflex are Aδ-fibers (Ertekin et al., 1975), it seemed that Aδ-fibers were stimulated by magnetic stimulation. However, due to weaker effects on the skin, the reflex could be observed with lower pain by magnetic stimulation than by bipolar. Some disadvantages of magnetic stimulation include the following: the device is uncommon, its maximum output is relatively weak and sometimes below the reflex threshold, the probe is large, and the device is noisy.

With respect to monopolar stimulation, a weaker pain sensation would be due to the lower current density on the skin caused by the wide electrodes. Unlike magnetic stimulation, monopolar stimulation can be performed with a common electrical neurostimulator. Monopolar stimulation usually has the disadvantage of being less focused than bipolar stimulation (Kombos et al., 1999; Gomez-Tames et al., 2018). However, in this study, it has merits in that the influence of the attachment site is relatively small and the experiment is more reproducible, because the sural nerve generally does not contain motor fibers (Amoiridis et al., 1997) and it is difficult to objectively identify its exact location. In addition, this method could reduce extra pain in the skin caused by stimulating the wrong position displaced from the nerve. Although the tibial nerve afferents in the sole may be stimulated and contribute to the flexion reflex (Ellrich and Treede, 1998), the effect is considered small if present as a large area electrode is used as an anode.

Although the lack of a significant difference of the reflex magnitude among the three stimulation methods may have been due to insufficient detection power with the small sample size, the present results clearly showed that wind-up of the flexion reflex could be observed with all three stimulation methods and magnetic and monopolar methods resulted in fewer pain scores. Regarding the best stimulation parameters, long measurement times may add uncertain effects, such as facilitation by negative emotions (Fragiotta et al., 2019), suppression by descending inhibitory controls (Gozariu et al., 1997; Ruscheweyh et al., 2011), or alteration of RIII reflex threshold by central sensitizations (Leone et al., 2021). Stimulation with 5–10 pulses at 4 Hz seems most suitable because wind-up effect was more obvious at higher frequencies, up to 4 Hz. To observe the later slope (Figure 3), stimulation at approximately 2 Hz would be better due to pain tolerance. As 0.9 times the threshold was not sufficient for some participants to observe wind-up, 1.0 times, which was sufficient for all, seems appropriate. Therefore, a practical method for observing the wind-up of the flexion reflex is to use 5–10 consecutive stimuli at 4 Hz or 10–15 stimuli at 2 Hz using single pulses at the reflex threshold with monopolar stimulation.

One important aspect of wind-up is the involvement of *N*-methyl-D-aspartate (NMDA) receptors in dorsal horn neurons (Herrero et al., 2000; Fossat et al., 2007; Aby et al., 2019) and wind-up of the flexion reflex in humans is indeed inhibited by the NMDA receptor antagonist (Arendt-Nielsen et al., 1995; Guirimand et al., 2000). Therefore, quantification of wind-up in individual subjects would be useful for evaluation of NMDA receptor function and chronic pain diagnosis or psychiatric disorders. Further investigations are needed to clarify how NMDA receptors are involved in the two mechanisms described.

## Conclusion

The wind-up effect can be observed using all of the three methods. Pain ratings for magnetic and monopolar stimulations were significantly lower than those for bipolar stimulation; however, wind-up effects for flexion reflex and pain did not differ. Although wind-up is thought to be mainly mediated by C-fibers, particularly in animals, there is another mechanism that does not require C-fibers. Observation of wind-up using monopolar stimulation would be useful for investigating short-term plasticity of the nociceptive pathway.

## Data Availability Statement

The raw data supporting the conclusions of this article will be made available by the authors, without undue reservation.

## Ethics Statement

The studies involving human participants were reviewed and approved by the National Institute for Physiological Sciences, Okazaki, Japan (approval number: 21A001). The patients/participants provided their written informed consent to participate in this study.

## Author Contributions

TT, SS, and KI: study concept and design, patient recruitment and data collection, data analysis and interpretation, and manuscript preparation. TK, NT, and MN: study concept and design, data collection, data analysis and interpretation, and manuscript preparation. KN: study concept and design, data analysis and interpretation, and manuscript preparation. All authors contributed to the article and approved the submitted version.

## Conflict of Interest

The authors declare that the research was conducted in the absence of any commercial or financial relationships that could be construed as a potential conflict of interest.

## Publisher’s Note

All claims expressed in this article are solely those of the authors and do not necessarily represent those of their affiliated organizations, or those of the publisher, the editors and the reviewers. Any product that may be evaluated in this article, or claim that may be made by its manufacturer, is not guaranteed or endorsed by the publisher.
