# Supplementary material for: A Minimally Invasive Method for Observing Wind-Up of Flexion Reflex in Humans: Comparison of Electrical and Magnetic Stimulation
Source: Front Neurosci. 2022 Feb 23;16:837340. doi: 10.3389/fnins.2022.837340 (PMC8904398; doi:10.3389/fnins.2022.837340)
Supplement: Supplementary file 1 [file Image_1.pdf]

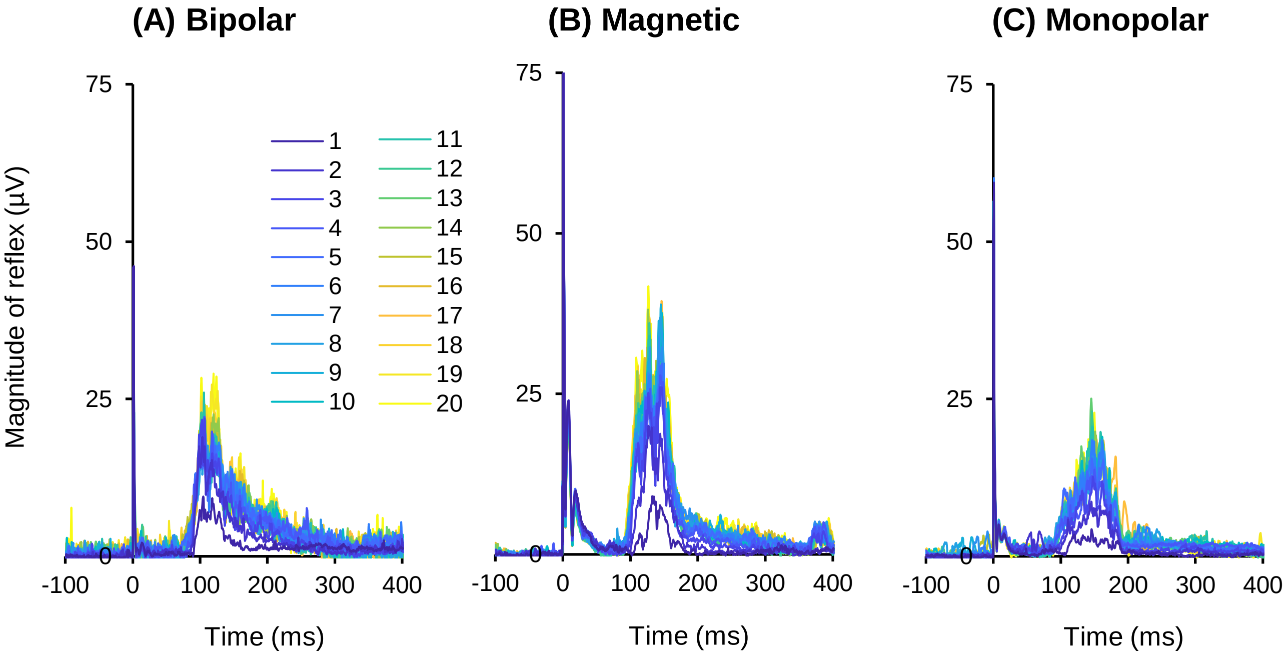

Supplementary Figure S1. Averaged electromyography waveforms across 11 participants in Experiment 1. The waveforms elicited by a series of 20 consecutive stimulations at the reflex threshold at 2 Hz by bipolar (A), magnetic (B), and monopolar (C) stimulation
