# Supplementary material for: A Minimally Invasive Method for Observing Wind-Up of Flexion Reflex in Humans: Comparison of Electrical and Magnetic Stimulation
Source: Front Neurosci. 2022 Feb 23;16:837340. doi: 10.3389/fnins.2022.837340 (PMC8904398; doi:10.3389/fnins.2022.837340)
Supplement: Supplementary file 2 [file Image_2.pdf]

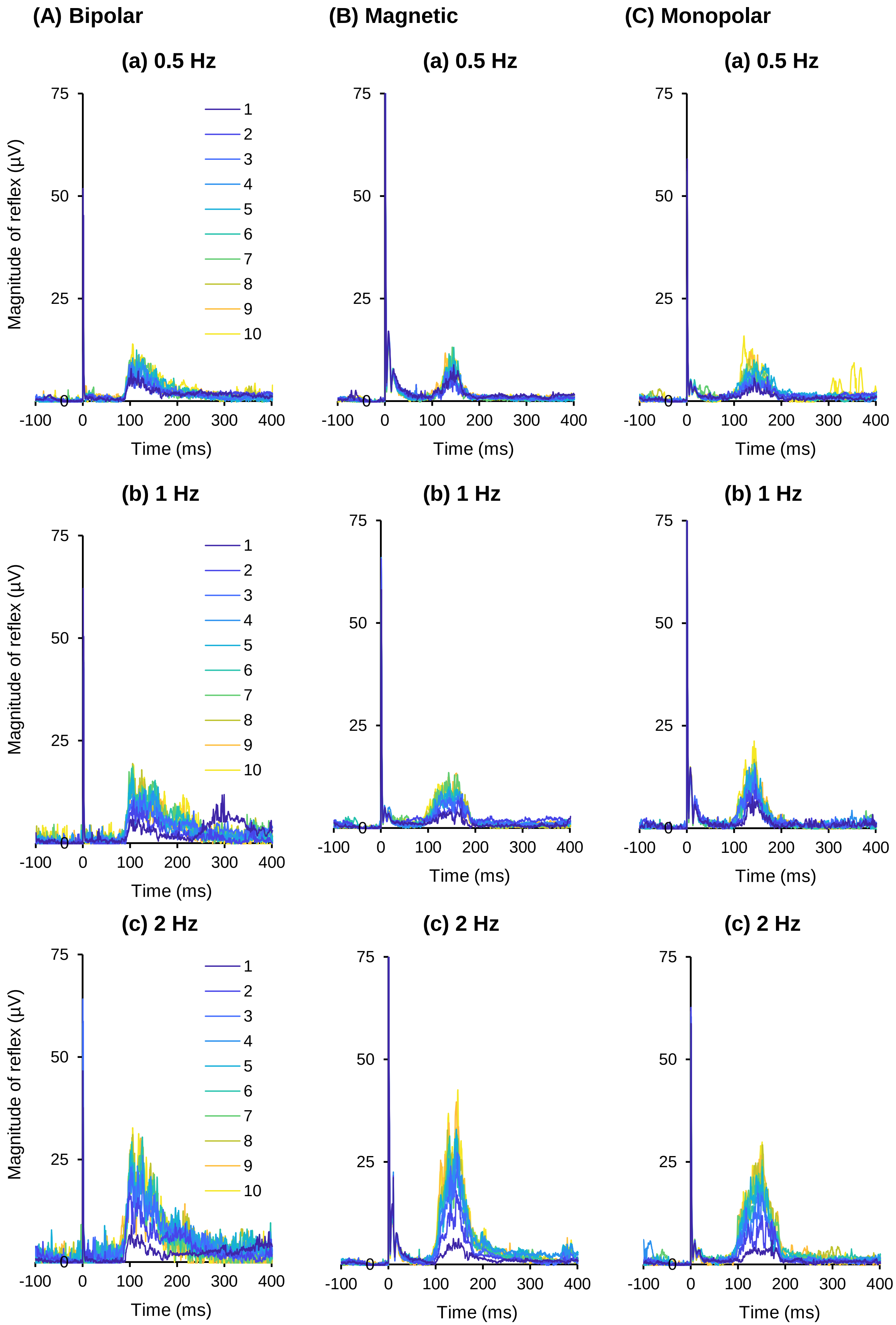
(A) Bipolar
(B) Magnetic
(C) Monopolar
(a) 0.5 Hz
(b) 1 Hz
(c) 2 Hz
Magnitude of reflex (μV)
Time (ms)
1
2
3
4
5
6
7
8
9
10

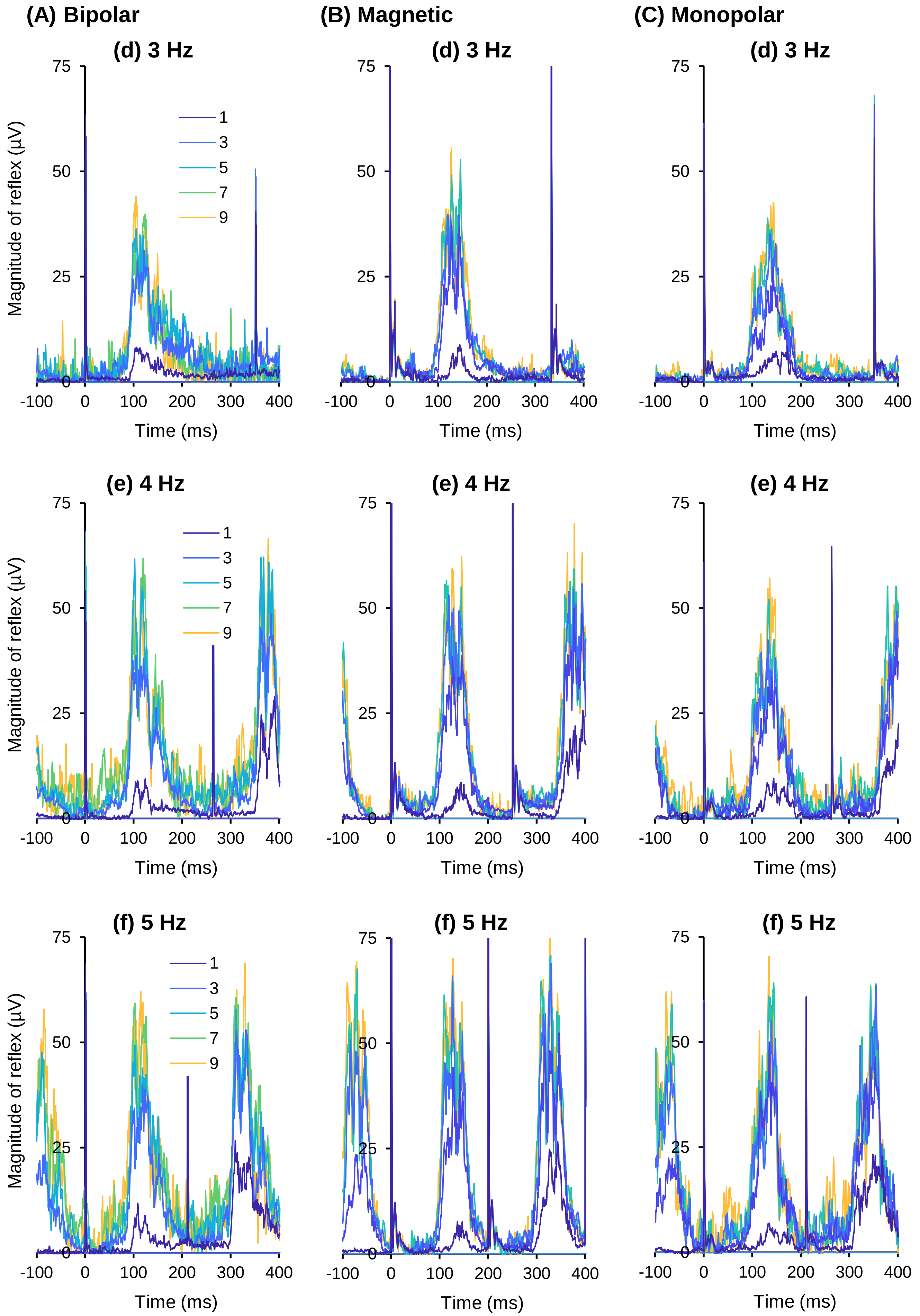


**Supplementary Figure S2.** Averaged electromyography waveforms across 11 subjects in Experiment 2. The waveforms elicited by a series of 10 consecutive stimulations at the reflex threshold by bipolar (A), magnetic (B), and monopolar (C) stimulation, at 0.5 (a), 1 (b), 2 (c), 3 (d), 4 (e) and 5 (f) Hz
