# Supplementary material for: A Minimally Invasive Method for Observing Wind-Up of Flexion Reflex in Humans: Comparison of Electrical and Magnetic Stimulation
Source: Front Neurosci. 2022 Feb 23;16:837340. doi: 10.3389/fnins.2022.837340 (PMC8904398; doi:10.3389/fnins.2022.837340)
Supplement: Supplementary file 3 [file Image_3.pdf]

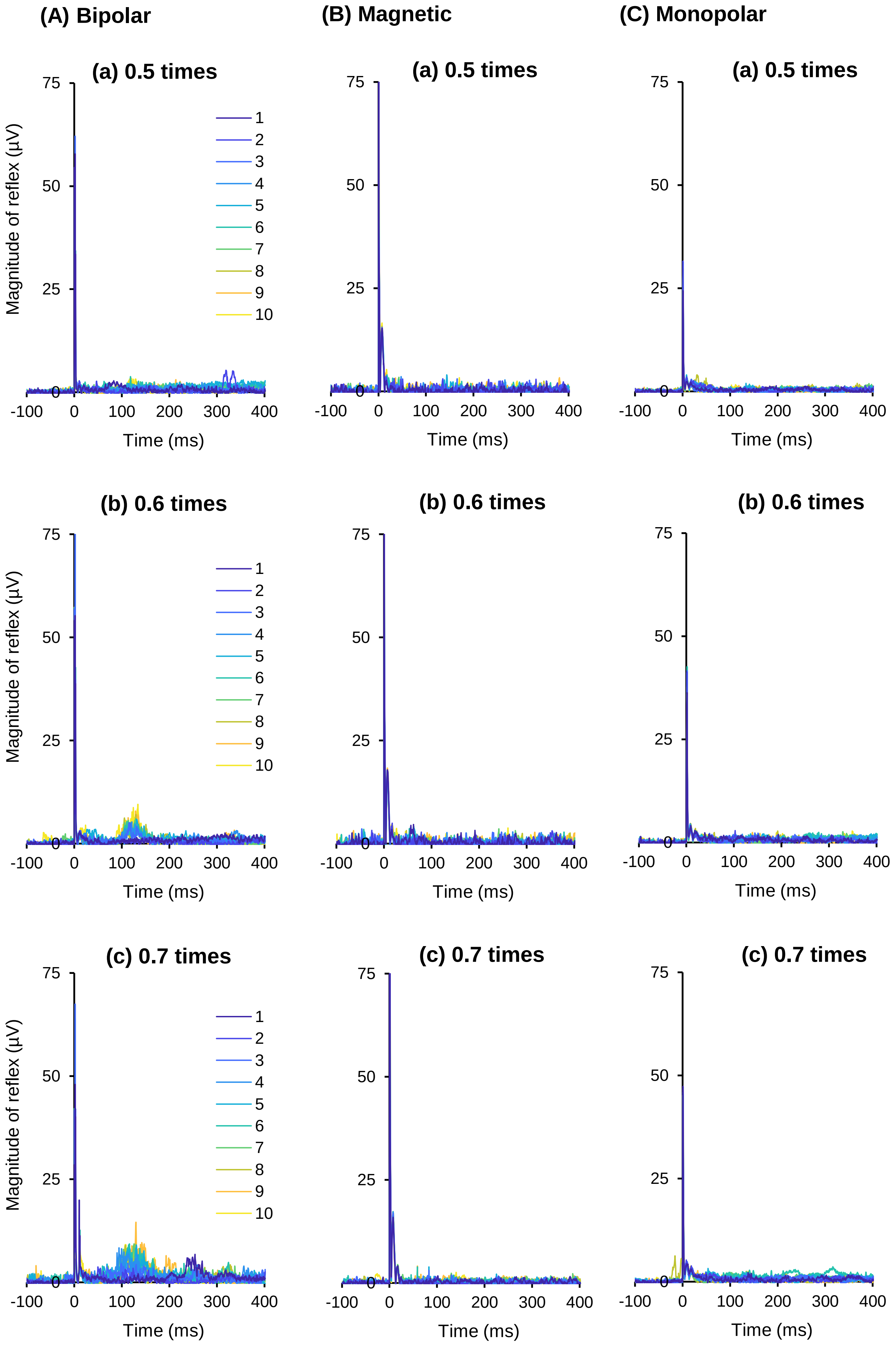
(A) Bipolar
(B) Magnetic
(C) Monopolar
(a) 0.5 times
(b) 0.6 times
(c) 0.7 times
Magnitude of reflex (µV)
Time (ms)
1
2
3
4
5
6
7
8
9
10
75
50
25
0
-100
0
100
200
300
400

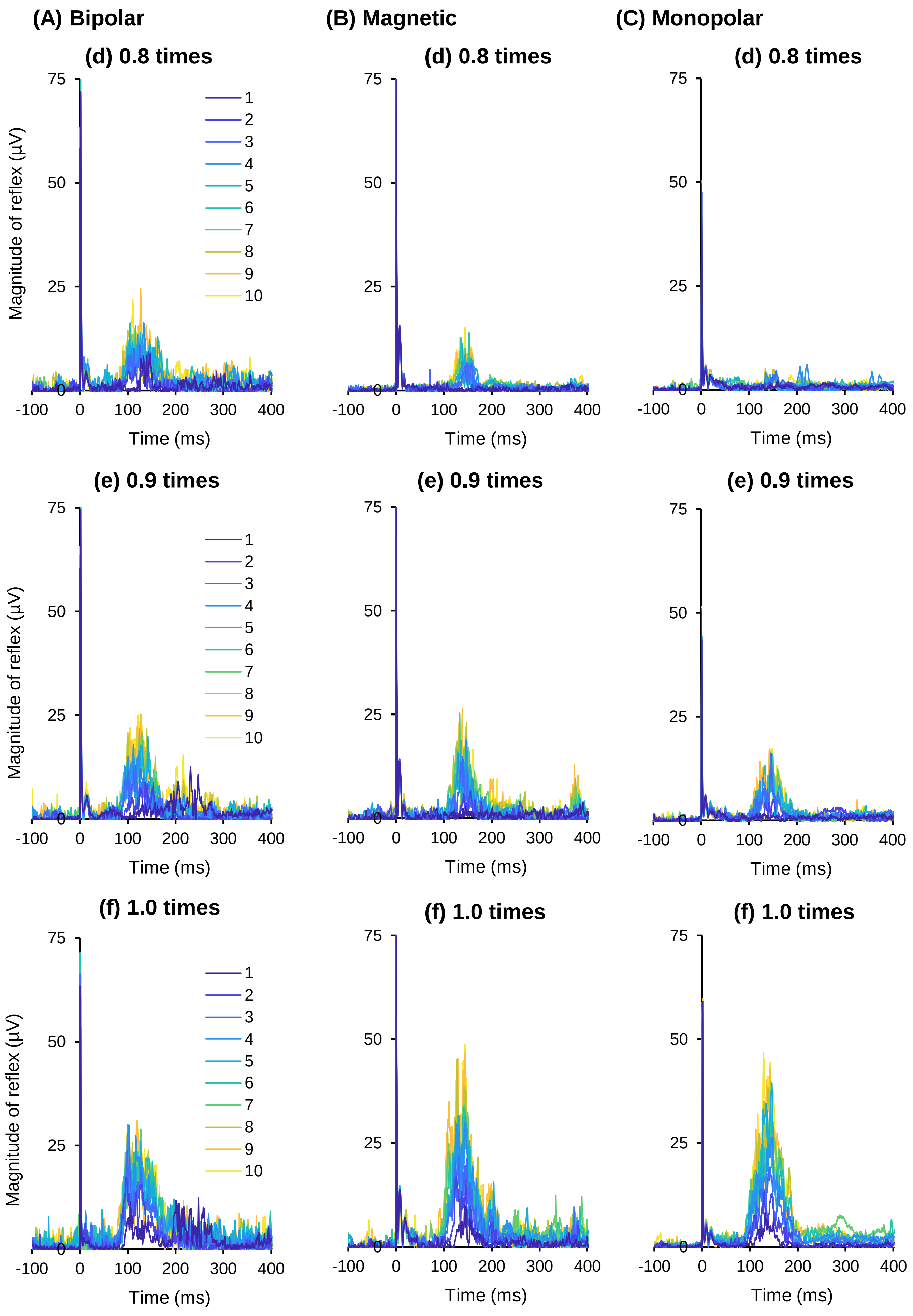

Supplementary Figure S3. Averaged electromyography waveforms across 11 subjects in Experiment 3. The waveforms elicited by a series of 10 consecutive stimulations at 2 Hz, by bipolar (A), magnetic (B), and monopolar (C) stimulation, at 0.5–1.0 (a–f) times the reflex threshold
